# Civil Society’s Evidence-Generating Role for Health Policy Decisions: A Thematic Analysis of a Healthcare Information for All (HIFA) Community Online Discussion

**DOI:** 10.34172/ijhpm.8701

**Published:** 2025-07-12

**Authors:** Unni Gopinathan, Tarry Asoka, María Eugenia Aponte-Rueda, Genevieve Cecilia Aryeteey, Esha Ray Chaudhuri, Meena Cherian, Praveen Devarsetty, Claire Glenton, Augustina Koduah, Tripti Gupta, Simon Lewin, Jacinta Nzinga, Velisha Ann Perumal-Pillay, Ravi Ram, Fatima Suleman, Goran Abdulla Zangana, Neil Martin Pakenham-Walsh

**Affiliations:** ^1^Cluster for Global Health, Division for Health Services, Norwegian Institute of Public Health, Oslo, Norway.; ^2^Centre for Epidemic Interventions Research, Division for Health Services, Norwegian Institute of Public Health, Oslo, Norway.; ^3^Independent Consultant, Port Harcourt, Nigeria.; ^4^Venezuelan Breast Cancer Research and Education Foundation (FUVEICAM), Caracas, Bolivarian Republic of Venezuela.; ^5^School of Public Health, College of Health Sciences, University of Ghana, Accra, Ghana.; ^6^Independent Patient Advocate, Calgary, AB, Canada.; ^7^Geneva Foundation for Medical Education & Research (GFMER), Geneva, Switzerland.; ^8^George Institute for Global Health, Hyderabad, India.; ^9^Department of Health and Functioning, Western Norway University of Applied Sciences, Bergen, Norway.; ^10^School of Pharmacy, College of Health Sciences, University of Ghana, Accra, Ghana.; ^11^External Consultant, National Health Systems Resource Centre, New Delhi, India.; ^12^Department of Health Sciences Ålesund, Norwegian University of Science and Technology (NTNU), Ålesund, Norway.; ^13^Health Economics Research Unit, KEMRI Wellcome Trust Research Programme, Nairobi, Kenya.; ^14^Discipline of Pharmaceutical Sciences, School of Health Sciences, College of Health Sciences, University of KwaZulu-Natal, Durban, South Africa.; ^15^Madhira Institute, Nairobi, Kenya.; ^16^Discipline of Pharmaceutical Sciences, School of Health Sciences, University of KwaZulu-Natal (Westville Campus), Durban, South Africa.; ^17^National Health Service (NHS) Lothian, Edinburgh, UK.; ^18^Healthcare Information For All, Chipping Norton, UK.

**Keywords:** Health Policy-Making, Civil Society, Public Participation, Experiential Evidence, Communities of Practice

## Abstract

Civil society actors are widely recognized for advocating the public interest in health policy. However, their role in contributing different types of evidence to inform policy is less explored. To explore this topic, members of the Healthcare Information for All (HIFA) online forum and the *Supporting Inclusive and Accountable Health Systems Decisions for Universal Health Coverage* (SUPPORT-SYSTEMS) research project conducted a four-week online discussion. The discussion focused on defining civil society, its role in health policy, the types of evidence it provides, and how this evidence is used and valued. Weekly focal questions encouraged HIFA members to share experiences of civil society engagement and the use of evidence in health policy-making. The thematic analysis identified four key messages. First, defining civil society requires critical reflection, as actors differ significantly in their interests, political ties, and influence. These distinctions affect how representative their evidence is and whether it reflects vested interests. Second, policy-making structures can support meaningful civil society participation, thereby strengthening the use of evidence and the legitimacy of policy decisions. Third, civil society provides valuable local and tacit knowledge that complements scientific evidence, though safeguards are needed to prevent bias or misrepresentation. Fourth, political economy factors—such as power imbalances, gatekeeping, and funding constraints—shape the influence of civil society evidence on policy. Overall, the discussion highlighted the diverse roles civil society can play in health policy and the importance of institutional mechanisms to support responsible evidence use. Thematic discussions in communities of practice (CoPs) like HIFA offer a dynamic and inclusive approach to engaging stakeholder knowledge in research projects.

## Introduction

 The past two decades have seen a surge in research and development in evidence-informed health policy-making.^1,2^ Much of this research has explored how policy-makers can be supported in using the best available knowledge when effectively and equitably introducing new health interventions, and financing, governing and organizing health systems.

 Civil society actors play a crucial role in representing the public’s interests, needs, and values in health systems, particularly for marginalized groups facing barriers to healthcare access and participation in decision-making.^3^ Less recognized, however, is their role in generating evidence for health policy decisions—an area that remains underexplored compared to their contributions to advocacy, health service delivery and the promotion of human rights and equity values.^4,5^

 Two factors complicate the discussion about how evidence from civil society can contribute to well-informed health policy decisions. First, varying interpretations of what qualifies as “evidence.” “Evidence” may be defined narrowly as research-based knowledge or more broadly as also encompassing other forms of knowledge, such as tacit or experiential knowledge—areas where civil society actors may be uniquely positioned to make vital contributions.^2,6-8^ Second, there also exists varying definitions and interpretations of what constitutes “civil society.”^9^ For the purpose of this article, we define civil society actors broadly as ranging from international, national or local non-governmental organizations to community-based organizations, loosely organized social movements or groups of individuals that are convened, for instance, through village assemblies or townhall meetings.^5^

 There are several reasons why gaining better insights into the role of civil society in generating evidence for health policy-making can be valuable. First, different assumptions may exist about what constitutes “civil society actors” and their “evidence,” and we can further our understanding of these terms from those with direct experience in these processes. Second, these explorations can draw attention to an underused source of knowledge, clarify the complementary role this evidence can play alongside scientific evidence, and identify factors that either impede or facilitate its use. Third, exploring experiences can foster learning, both among civil society actors and between civil society actors and policy-makers, on how to use this evidence more effectively.

 Online communities of practice (CoPs) can provide a valuable source for connecting theoretical concepts with real-world experiences.^10^ One such CoP is Healthcare Information for All (HIFA, https://hifa.org/), with members worldwide from diverse professional backgrounds including health workers, librarians, publishers, researchers, policy-makers, and civil society representatives. HIFA’s over 10 000 members interact on multilingual forums, sharing experiences to improve the availability of reliable healthcare information for health workers, the public, and policy-makers, and thereby improve quality of care.^11^ Insights from HIFA forum members have previously informed other research projects,^10^ as well as several World Health Organization (WHO) guidelines, including guidance on optimizing health worker roles for maternal and newborn health through task shifting^12,13^ and guidance on health system support to optimize community health worker programmes.^14^ In this short communication, we present key findings from a HIFA thematic discussion aimed at exploring HIFA members’ experiences with the generation and use of evidence from civil society.

## Methods

###  Context and Rationale for Implementing a Thematic HIFA Discussion

 The discussion was implemented in the context of a larger research project, *Supporting Inclusive and Accountable Health Systems Decisions for Universal Health Coverage *(SUPPORT-SYSTEMS), a four-year initiative funded by the Research Council of Norway.^15^ One of the project’s major goals is to conduct a Cochrane qualitative evidence synthesis on using civil society evidence in health policy processes.^9^ Preparing the review protocol highlighted the need to better define key concepts such as “civil society” and “evidence” and to understand how civil society actors and policy-makers perceive these concepts. The HIFA forum was identified as a suitable platform for eliciting these perspectives. We chose the HIFA forum for its global reach and participants with diverse geographic and professional backgrounds; including patient representatives, civil society actors, healthcare providers, and policy-makers from ministries and multilateral organizations. Additionally, the four-week format allowed for extended engagement, fostering continuous interaction on shared insights. HIFA’s prior experience with thematic discussions meant that many participants were already familiar with this approach and understood the type of insights such discussions aim to generate.

###  Approach: Establishment of a HIFA Working Group, Formulating Questions and Implementing the Thematic Discussion

 In March 2022, HIFA established a working group consisting of 12 volunteers recruited from existing HIFA members and members of the SUPPORT-SYSTEMS research project team.^16^ The project group convened twice to formulate, refine and agree on questions to structure the four-week HIFA online discussion. The weekly questions for the thematic discussion were formulated based on an initial concept note developed to inform a systematic review,^17^ followed by discussions among HIFA working group members to define the questions they believed would be most useful for eliciting valuable experiences on the use of evidence from civil society. The questions were focused on the nature of civil society and its role in health policy processes; the types of evidence presented in decision-making forums; how this evidence is used and valued; and promising practices for using evidence from civil society in health policy processes (See Table 1 for exact questions posed to the forum). The online discussion was held between May 9 and June 6, 2022. The thematic discussion was open to all HIFA members. Members were encouraged to contribute, react, and engage in discussions, while facilitators provided comments to prompt participation from those with relevant experience. HIFA members have consented to having their profiles and posts publicly available—with an option to withdraw—with the HIFA forum, under the Global Healthcare Information Network, serving as the General Data Protection Regulation-compliant data controller.^18^

**Table 1:** Weekly Questions Asked Per Week During the Thematic Discussion

**Week**	**Questions **
Week 1	What does civil society participation in health policy mean and why is it important to have civil society participation in health policy processes?
Week 2	Have you ever participated, either through a CSO or as an individual, in health policy processes at a national or sub-national level? What was your experience?
Week 3	Can you share examples of the role of CSOs in policy-making at national or sub-national levels?
Week 4	What do you think are the different types of evidence that civil society can provide, that otherwise would not be considered? What are the main drivers and barriers to uptake and use of such evidence?

Abbreviation: CSO, civil society organization.

###  Data Collection and Analysis 

 Four members of the SUPPORT-SYSTEMS HIFA working group (UG, TA, TG, and NPW) shared responsibility for monitoring and keeping track of the discussion and producing (1) a full compilation of the contributions; (2) a long edit; and (3) a thematic analysis of the contributions. The long edit was a verbatim chronological compilation of every contribution made, organized by each week. This document was used by the lead author to conduct a thematic analysis. The analytic approach involved reading and familiarizing with the contributions, categorizing the content of each contribution under distinct codes and further refining and combining codes into major themes.^19,20^ The preliminary themes were shared and discussed with the other members of the working group during one virtual meeting before further refining the categories. No a priori organizing theoretical framework was used to structure and interpret the data. For contributions that provided citations to support their claims, these citations have been included when presenting the findings.

###  Reflexivity Statement

 The HIFA thematic discussion was initiated by researchers from the SUPPORT-SYSTEMS research project (UG, GCA, CG, AK, SL, and JN) and HIFA. Contributors to the working group and the discussion were invited to participate in the interpretation of the thematic analysis and to co-author the article. Consequently, many of the contributions to the discussion forum, which underlie the thematic findings, came from the co-authors of this piece. Thus, a reflexive stance and positioning were embedded in the analysis, ensuring that alternative interpretations and rival findings were explored to avoid presenting a biased view.

## Results

###  Descriptive Overview of the Contributors

 Fourteen HIFA members made a total of 80 contributions to the online discussion. These 14 contributors were from 10 countries: Canada, Croatia, India, Iraq, Nigeria (3), Norway (2), South Africa, Tanzania, the United Kingdom (2), and Venezuela. Their professional backgrounds spanned healthcare, civil society organizations (CSOs), research, and policy, with expertise in areas such as clinical governance, pharmaceutical policy, health systems strengthening, and civil society advocacy. Seven contributors had extensive experience working within civil society networks or non-governmental organizations and/or participating in multi-country civil society coalitions to advocate for universal health coverage. Three contributors had their main experience from research institutions, focusing on evidence-informed policy-making in a wide set of areas, including pharmaceutical policy, universal health coverage and health systems strengthening. Five had substantial experience in frontline healthcare and service delivery, including for underserved populations. Two contributors had engaged in policy-making by serving in formal ministry roles or technical advisory committees informing health policy decisions at the national or sub-national level, focused on national health strategies, implementation of universal health coverage policies, and clinical governance structures. In the following, we discuss key themes identified from the thematic analysis of these contributions.

####  Defining the concept of civil society needs critical evaluation and its interpretation should be sensitive to local context

 In exploring the evidence-generating role of civil society, contributors stressed the need to critically examine which actors are included in definitions of civil society, highlighting that historical and socio-political contexts shape these definitions. Contributors advocated for a flexible understanding of civil society rather than following a rigid definition, considering the types of actors, their level of operation, and the power they held. Frequently used terms like “citizen engagement,” “community involvement,” “community participation,” and “empowerment” were argued to need critical evaluation in terms of the extent to which these truly represent the interests of different groups in society. One contributor expressed this as: “CSOs are, again, not a homogeneous group of organisations just like the communities or the population groups they serve” (paediatrician and public health practitioner, India). Another contributor highlighted that “organizations” under the umbrella of “civil society” may take multiple forms, ranging between activist groups, charities, non-governmental organizations, non-profit organizations, private voluntary organizations, social enterprises, social movement organizations, and/or voluntary associations.

 It was questioned whether actors that fulfilled the formal definition of “civil society” but primarily represented powerful interests through their administrative, professional and political power truly could fit in this conventional concept. To illustrate this point, one contributor with experience from Iraq described how “civil society organisations (and groups) can be categorised into two main groups in Iraq; one that are truly grassroot and representative of a section of the society (few and rare and not very influential) and ones that are affiliated with political parties and/or linked in one way or another to the government (more powerful)” (medical doctor, Iraq/United Kingdom). Similarly, a contributor from Venezuela pointed out that “the CSOs are a heterogeneous group of organizations as the communities or the population groups whose scope of action will depend on the context they serve” (clinician and CSO activist, Venezuela). Further examples presented in the next section underscore that civil society’s meaning and influence are shaped by the historical and socio-political context.

####  Institutional structures can create a pathway for broad civil society involvement, collective action, and legitimacy of health policy processes

 Examples from different regional and socio-political settings shared by contributors highlighted the importance of strong institutional structures that can facilitate broad civil society involvement in shaping health policy, while also revealing systemic constraints that can hinder the effective use of their evidence. From Venezuela, one contributor referenced literature from Latin America highlighting how structural conditions shape CSO influence. One example was from Uruguay, where strong political backing, financial and technical support and collaboration with transnational tobacco control networks empowered local CSOs to work with the government to defend tobacco control policy against legal threats from Philip Morris International.^21^ At this particular point in time in Uruguay, the tobacco control efforts where characterized by strong alliances between public health experts and reform-minded political leaders and progressive policy-makers in Uruguay were receptive to evidence-based inputs from civil society.^21^ Another contrasting example from Argentina reported how mental health user associations faced power imbalances, professional gatekeeping, and historical disempowerment; limiting their ability to influence care and treatment policy.^22^ Important to Argentina’s context was the enactment of a mental health law in 2010, whereupon mental health users’ associations increased in response to legal mandates and local organizational structures that fostered direct inclusion of mental health users in oversight roles. However, a professional culture that had emphasized medical authority over patient participation restricted the agency of mental health users and reinforced their marginalization within the system.^22^

 Another example that was given from West Africa (The Gambia, Ghana, Liberia, Nigeria, and Sierra Leone) was the regional Mental Health Leadership and Advocacy Programme (mhLAP). Organized over a 10-year period, this programme enabled the participation of community groups, patient associations and health professional associations in shaping mental health policy in these countries.^23^

 According to one contributor, a key outcome of the mhLAP was the establishment of National Stakeholder Councils to support broader participation in mental health policy. These councils had representation from mental health professionals, representatives of service user and caregiver organisations, officials of relevant government departments and agencies, leaders of non-governmental organizations with an interest in mental health or human rights issues, and media practitioners. The contributor described how the establishment of such a structure created a pathway for civil society actors to shape policy and legislation on mental health, and underscored the importance of permanent structures that facilitate civil society participation in health policy formulation:

 “*The Stakeholder Councils that were activated in each country became effective and credible voices in all of the participating countries, as they were actively engaged in efforts to develop policy and legislation and championing community outreach programmes. For example, mhLAP was noted to have made notable contributions to the processes leading on to the adoption of the Mental Health Policy in Nigeria and the Mental Health Act in Ghana, where the project provided leadership to civil societies and other stakeholders involved” *(Health sector consultant, Nigeria).

 The establishment of national stakeholder councils with broad representation also helped foster the legitimacy of the policy-making process.

 “*The Country Facilitators, as well as these graduates, are guided by priorities set by a wide coalition of stakeholders in the country, creating a unified message, with the legitimacy that comes from such a broad-based constituency. Indeed, the expectation from the leadership course participants is that they become informed advocates for mental health service development in their respective countries” *(Health sector consultant, Nigeria).

 Reflecting on observations from Iraq, a contributor raised whether a civil society advisory committee within the Ministry of Health could have provided a structured platform for engagement about Iraq’s policy, which was about government facilities charging private market fees for publicly funded services after 1PM. Despite CSOs presenting strong evidence on the financial burden of this “semi-private” system—including its impact on impoverishment and failure to meet universal health coveragegoals—decision-making was dominated by financial constraints and powerful interest groups, particularly health professionals advocating for the policy. Without an institutionalized mechanism, the contributor argued that the advocacy efforts and evidence use by CSOs were reactive and lacked sustained influence on policy-making.

 Overall, these examples illustrated that institutional structures that provide space, resources, and safeguards for CSOs are essential for their meaningful participation in health policy. Without these conditions, participation remains constrained, limiting efforts to promote the public good and weakening the legitimacy of policy-making processes.

####  Civil society actors are uniquely positioned to inform policies with tacit and local evidence, but this evidence must be used carefully to avoid misrepresentation and bias 

 Using WHO’s recent publication on evidence, policy and impact as a starting point,^2^ one contributor suggested that policy-makers’ needs are usually met by four types of evidence: (1) scientific and codified evidence; (2) tacit evidence; (3) global evidence assembled for example through a systematic review; and (4) local evidence. The empirical examples shared by contributors suggested that “evidence” from a civil society perspective can include information that can be placed under each of these categories but also that these groups are overlapping, since both scientific and tacit evidence can be global and local in nature (Table 2).

**Table 2:** Categories of Evidence That Civil Society Actors Can Contribute With

	**Global**	**Local**
Scientific evidence	Primary research summarized in a systematic review	Conducting local primary research, through for example surveys or interviews, or interpreting existing primary research
Tacit evidence	Cross-country experiential knowledge on illness management, policy effects, and health system challenges shared by civil society networks, advocacy groups, and international organizations Informal knowledge from global practitioners and policy-makers on implementing and adapting health interventions across diverse contexts	Experiential knowledge about illness or disease Knowledge about effects of legislation and policy on people Voices of the population or specific groups

 It was expressed that the value placed on these different sources may not necessarily follow the typical evidence hierarchies, and alternative ways of thinking about evidence for informing health policy decisions could be helpful. For example, a contributor highlighted how the Treatment Action Campaign—a prominent South African advocacy organization promoting affordable HIV/AIDS treatment and broader healthcare rights—framed multinational pharmaceutical companies’ excessive pricing of essential medicines as human rights violations.^24^ Although such views are not typically included in conventional evidence frameworks, this was perceived as a profoundly compelling piece of evidence.

 Civil society actors, through their deep community ties, diverse perspectives, and continuous interaction with health systems, was seen to uniquely hold tacit knowledge that can fill evidence gaps. One contributor described tacit knowledge as the lived experiences of individuals who have valuable insights to share, enabling collective learning. This interaction can lead to personal insights, such as applying a new approach to one’s own context, and occasionally, it may generate new collective insights. Contributors also saw HIFA as a civil society forum that could share tacit as well as explicit knowledge, where the latter could take the form of publications and reports. While conducting research was a way civil society can generate scientific evidence, contributors highlighted that small CSOs typically do not have the capacity nor remit to conduct academic research using rigorous methods. To overcome this shortcoming in capacity, civil societies could collaborate with research institutions.

 Contributors expressed that the involvement of civil society in health policy processes may not necessarily ensure that the best available evidence informs decisions. For example, civil society actors may promote evidence that is aligned with their own or political agenda and objectives and thereby risk contributing with a cherry-picked and biased assessment of the evidence base. Participants, therefore, considered it important to explore which processes are most effective in empowering decision-makers to use evidence from civil society while ensuring it is used carefully to avoid misrepresentation and bias.

####  Political economy factors may impact on the uptake and influence of evidence from civil society

 A sound understanding of the political economy factors—the influence of politics, power and resources on policy-making—can help us explore why civil society is able to successfully inform and influence health policy in some areas while not in others.^25^ A contributor highlighted that differences in the sources of power held by civil society actors could affect the impact of their evidence on policy. For example, health professional associations, like medical associations, are civil society actors that hold administrative power through their organizational structures and ability to engage with government processes, as well as professional power through their expertise to shape practices and influence policy. Other kinds of civil society actors may hold other types of power, for instance through varying degrees of links to political parties and good access to decision-making processes.

 An example of political economy dynamics influencing the use of evidence from civil society was the earlier example shared from Argentina, where mental health user associations sought involvement in policy-making through institutional channels established by the 2010 National Mental Health Law, such as the *Review Body* (*Órgano de Revisión*) and the *Honorary Consulting Council* (*Consejo Consultivo Honorario*).^22^ However, their ability to participate autonomously was constrained by entrenched power structures. Professional conflicts, particularly between psychiatrists resisting deinstitutionalization and psychologists supporting the reform, pulled user associations into existing power struggles instead of allowing them to advocate independently. Historical disempowerment was said to further limit their influence, as many associations relied on professionals or family members for leadership, reinforcing paternalistic structures. Economic constraints and shifts in government support also apparently weakened their capacity for sustained advocacy. As a result, the contributor argued that user organizations remained dependent on alliances with more powerful actors, preventing them from fully exercising the participatory role envisioned in the reform.

 The case of Iraq’s policy allowing public facilities to charge private market fees after 1 pm, mentioned earlier, further highlights how power dynamics shape the use of evidence in policy-making. While “evidence” may support one course of action, decision-makers may be guided by competing values and interests. A contributor shared an experience of civil society efforts to reverse this policy in the Kurdistan Region, where CSOs presented a range of evidence sources:

 “*A number of civil society organisations came together to advocate against this policy. We had meetings with policy-makers including the ministry of health and the health committee of the Parliament. We provided evidence regarding the negative impact of such policy on people’s financial wellbeing. We offered real life examples of people who had to sell properties, lands, borrow money... etc to pay for healthcare. We cited World Bank research that suggested that out of pocket healthcare expenditure was one of the main causes of impoverishment in Iraq”* (Medical doctor, Iraq/United Kingdom).

 Despite the strength of this evidence, the government upheld the policy. The contributor pointed to power imbalances as a key factor, particularly the influence of doctors and nurses who supported the policy and the opposition of strong interest groups to alternative revenue measures, such as taxes on tobacco, alcohol, and sugar. This case illustrates how entrenched power structures and economic interests can override evidence in decision-making. At the same time, the contributor noted that civil society’s presence and advocacy likely tempered some of the policy’s inequitable effects, underscoring the need for institutionalized mechanisms that can counterbalance the influence of vested interests in health policy-making.

## Discussion

 Evidence-informed policy-making is an area that has spurred extensive research on the generation, appraisal, and effective dissemination of research evidence to policy-makers and health policy-making processes. While definitions of evidence have varied, sometimes recognizing experiential and tacit knowledge, the literature has largely focused on scientific evidence.^9^ However, several global developments have drawn attention to the importance of considering evidence from civil society. WHO has advanced efforts on citizen engagement in evidence-informed policy-making, emphasizing the role of civil society actors in providing evidence, particularly through methods for eliciting citizen knowledge.^26^ There is also growing recognition of social participation as a driver of universal health coverage, reflected in WHO’s flagship report on social participation and the 2025 World Health Assembly resolution on social participation in health.^3,27^ WHO has also established the WHO Civil Society Commission to strengthen dialogue, foster collaboration, and systematically engage civil society in advancing public health and health-related SDGs at global, regional, and national levels.^28^ Against this background, our exploratory and participatory study highlights two key aspects: (1) the role of civil society in contributing evidence to health policy-making, demonstrating how its participation can support more responsive and equitable decisions, and (2) the value of engaging CoPs to refine key concepts and assumptions in research. We briefly discuss key insights from each of these in relation to existing literature.

 With respect to the use of evidence from civil society, the thematic discussion highlighted two valuable points. First, in appraising and using evidence from civil society to inform health policy-making, it is crucial to be cognizant of the need to adopt a pluralistic view of the civil society concept. Viewing civil society as a homogeneous group risks overlooking the diversity of actors, who may represent widely different, and at times conflicting, interests that do not always align with the public interest and equity goals. It is particularly crucial to distinguish between patient interests and broader public interests,^29^ and be aware of funding sources and the fact that civil society actors can take various forms, ranging from organized groups to individual participation in public forums. For example, Lim et al found that in South Korea’s reform that separated the prescribing and dispensing of pharmaceutical drugs, strong interest groups with more resources had greater influence than civic groups with limited technical expertise, leading to policy capture and unintended outcomes such as increased non-covered services and rising healthcare expenditures.^30^ The authors argued that civic groups should be strengthened relative to strongly organized interest groups in order to safeguard public interest in these processes.^30^ Recognizing these differences is crucial to ensuring that evidence from civil society is used in a legitimate and representative manner, preventing its distortion in policy-making in ways that favor vested interests, and ensuring that neglected or under-represented needs and perspectives are promoted. A key implication is that more participatory research is needed to develop tools that empower policy-makers to effectively and equitably engage with and use un-conventional sources of evidence provided by civil society actors.^31^

 Second, contributors shared examples demonstrating how institutional changes, such as the creation of permanent mechanisms for civil society deliberation or channels for soliciting evidence, play a critical role in enabling the use of civil society evidence in health policy-making. These insights align with recent literature and country case studies showing how institutional changes—or their absence—can facilitate or prevent civil society and public participation in health policy-making across diverse settings, including Ukraine, Philippines, Thailand, Gambia, South Africa, and Tanzania.^32^ Going forward, in-depth research should explore how policy-making can most effectively use different participatory models while ensuring inclusiveness and legitimacy, the conditions for their sustainability, and how to prioritize their implementation where most needed.

 Many research funders now require patient or public involvement in planning, conducting and disseminating the research that they fund; however, implementation of practices for patient or public involvement often falls short.^33^ Online CoPs like HIFA can serve an important role as a low-cost, accessible supplement to user participation in research. A key strength of the HIFA forum is its wide geographic representation and the inclusion of practitioners whose focus is not research alone. This includes health professionals involved in delivering health services, policy-makers from Ministries of Health and local government, as well as representatives from civil society. In our case, the HIFA discussion informed the development of a new Cochrane systematic review, making important contributions to how key concepts were defined.^9^ A similar approach was successfully used in another recent research project in which the HIFA forum was used to explore how health workers are currently using mobile phones, the circumstances that have prompted this use, and any self-initiated solutions that have emerged, and these insights helped inform a working definition of informal mobile phone use.^10^

 A key limitation of our discussion is the relatively limited number of participants who contributed with their comments and references. However, many contributors provided multiple inputs with in-depth information, enhancing the quality of the discussion with nuanced insights, which suggested a high level of engagement. A second limitation is that our questions took a global perspective without specifically eliciting experiences that may differ between high-income and low- and middle-income settings. This aspect could be explored through an evidence synthesis comparing findings from studies across different income levels and socio-political contexts. Finally, participation in the thematic discussion was self-selected among HIFA members, who may have a particular interest in civil society’s role in using evidence in health policy-making and a generally favorable view of its contributions. Therefore, it was especially important to include perspectives in the analysis that critically assessed the value of civil society evidence, such as concerns about misrepresenting evidence or using it to advance narrow interests.

## Conclusion

 The HIFA thematic discussion on inclusive and accountable decision-making processes for health systems strengthening and universal health coverage inspired a rich discussion highlighting the need to critically examine how civil society is involved in health policy processes, the mechanisms for achieving this goal and the value this involvement brings. Contributors shared empirical examples and literature covering a wide range of perspectives on these questions. The insights from the discussion informed the development of a protocol for a qualitative evidence synthesis on the use of evidence by civil society in health policy processes. Thematic discussions on CoPs like HIFA offer an innovative, low-cost, inclusive, and iterative approach to engage stakeholders’ experience and expertise across diverse geographic and professional backgrounds in research projects.

## Acknowledgements

 We are grateful to the contributions made by HIFA members to the thematic discussion that informed this manuscript.

## Ethical issues

 This work did not involve the collection of primary health data or other personal information from human subjects, and therefore did not require assessment by an ethics committee.

## Conflicts of interest

 Authors declare that they have no conflicts of interest.
